# Remarkable increase of musculoskeletal disorders among soldiers preparing for international missions – comparison between 2002 and 2012

**DOI:** 10.1186/s12891-019-2856-x

**Published:** 2019-10-12

**Authors:** Alexandra Halvarsson, Monika Seth, Matthias Tegern, Lisbet Broman, Helena Larsson

**Affiliations:** 1 Departments of Neurobiology, Care Sciences and Society, Division of Pjhysiotherapy, Karolinska Institutet, Huddinge, Sweden; 2 Allied Health Professionals Function, Karolinska University Hospital, Stockholm, Sweden; 3 Swedish Armed Forces, Medical Service, Stockholm, Sweden; 4 Department of Community Medicine and Rehabilitation, Umeå University, 901 87 Umeå, Sweden; 5 Swedish Armed Forces, Headquarters, Medical Services, Stockholm, Sweden

**Keywords:** Occupational health, Soldier, Risk factors, Workload, Musculoskeletal injuries/disorder

## Abstract

**Background:**

Musculoskeletal disorders (MSD) are common among soldiers and constitute the most common reason for discontinuing military service within different military populations worldwide. The aims of this study were to investigate the prevalence of musculoskeletal disorders in two cohorts, 10 years apart, in the Swedish Armed Forces, to explore differences between these cohorts and to determine associated factors with MSD.

**Method:**

Comparative cross-sectional study. Participants were recruited from the Swedish Armed Forces, i.e. soldiers preparing for international missions in 2002 and 2012. A total of 961 soldiers, 7% women, participated in the study.

Data were collected using the Musculoskeletal Screening Protocol (MSP), which includes questions regarding prevalence of MSD in ten anatomical locations (neck, upper back, low back, shoulders, elbow, hand, hip, knee, lower limb and foot). An additional five questions concern perceived self-rated health, i.e. how the respondent perceives their own physical body, mental health, social environment, physical environment and work ability.

**Results:**

Over a ten-year period, both point prevalence and one-year prevalence of MSD in any body part increased significantly, with point prevalence increasing from 7.1 to 35.2% (*p* < 0.001) and one-year prevalence from 27.9 to 67.9% (*p* < 0.001). The knee was the most common anatomic location for MSD in both cohorts. Across each anatomical location (neck, upper back, low back, shoulders, elbow, hand, hip, knee, lower leg and foot), both point prevalence (*p* < 0.039) and one-year prevalence (*p* < 0.005) increased significantly from 2002 to 2012.

Most soldiers reported good to excellent perceived health, i.e. self-perception of their physical body, mental health, physical and social environments, and work ability.

The odds of reporting one-year prevalence of MSD in any body part was 5.28 times higher for soldiers in Cohort 2012, 1.91 times higher in age group 31–40 and 2.84 times higher in age group 41 and above.

**Conclusions:**

The prevalence of MSD increased remarkably over a ten-year period among Swedish soldiers preparing for international missions. With increasing age as one risk factor, systematic monitoring of MSD throughout the soldiers’ careers and implementation of targeted primary-to-tertiary preventive programs are thus important.

## Background

Musculoskeletal disorders (MSD) constitute the most common reason for discontinuing military service within different military populations worldwide [1, 2], and the prevalence of these disorders is steadily increasing [1].

There is strong evidence within the military population suggesting that female gender, low aerobic fitness and endurance, extremes in flexibility, prior injury, cigarette smoking, participation in recreational sports activities, a history of prior limited physical activity, and older running shoes are risk factors for developing MSD [3, 4]. Specifically, the low back and knee joints are the most affected body regions [2, 5]. This is also true for Swedish Armed Forces (SwAF) soldiers deployed to Afghanistan, where a six-month MSD prevalence of 70% was found post-deployment. The most affected anatomic locations were the low back, shoulders, and lower extremities. A majority of the soldiers, however, reported that the MSD did not affect their daily work [6]. On the other hand, decreased work ability due to MSD has been found in studies of military populations in the United States, the Netherlands and Sweden [7–9]. The cause of reduced physical and mental working capacity could be, in part, attributed to repeated heavy work shifts and lack of recovery. Moreover, fatigue itself can induce increased risk for MSD, which further contributes to a reduced working capacity [7, 8].

MSD primarily caused, or made worse, by work and working environment is often described as work-related MSD [10]. Though the definitions may vary, work-related MSD are often multifactorial in origin and thus depend on a combination of risk factors, such as heavy work and lifting, awkward postures, previous complaints, work with high demands and with little control, physical inactivity, older age, female gender, high body mass index and tobacco smoking [11].

Studies have shown that the soldiers’ exposure to physical workload is higher today than in the early 2000s [8, 12]. The load of the soldiers’ equipment has steadily increased over the years. The increased weight is due to a combination of heavy weapons and combat equipment as well as new fighting equipment that the soldier must carry [13, 14]. In response to this increase in load, it has been suggested that the weight of combat equipment and the number of physically heavy working days in a row need to be monitored and limited, with the aim of increasing working capacity (i.e. the combat power) and longevity of soldiers [7].

While MSD imply an increased risk of discontinuing the basic military training, previous research within the SwAF has also found that this risk increases for conscripts who rated their health low [5, 15]. It is therefore of importance to identify the soldiers’ self-perceived health in order to gain an indication of how individuals will manage their work as a soldier. Moreover, it is also of great importance to investigate not only whether the prevalence of MSD has increased within the SwAF as previous studies [8, 12] have suggested, but also to assess the extent to which the increasing load of soldiers’ equipment in recent years relates to prevalence indices [13, 14].

The aim of this study was therefore to investigate the prevalence of musculoskeletal disorders (MSD), including pain and medically diagnosed injuries and self-perceived health, in two cohorts within the Swedish Armed Forces 10 years apart (2002 and 2012). The study furthermore aimed to explore any differences between the cohorts and to determine associated factors with MSD.

## Method

A comparative cross-sectional study design was used to study the prevalence indices of MSD in two cohorts of military personnel within the SwAF, 10 years apart. Participants were recruited from cohorts preparing for international deployment in 2002 and in 2012. All soldiers in both cohorts were asked to participate in the study.

In 2002, 810 soldiers were deployed. Of these, 204 did not follow the regular rotation schedule and could not be screened pre-deployment and were therefore excluded, leaving 606 included soldiers.

In 2012, 579 soldiers were deployed. Air force personnel and soldiers already enrolled in another study (*n* = 201) or denied participation (*n* = 23) were excluded. The remaining 355 soldiers from this cohort were included in the study. All soldiers perform a medical examination before entering the preparation phase before deployment, i.e. all soldiers are considered to be healthy and not suffering from any medical conditions.

Data were collected during the preparation phase, i.e. training period before the soldiers went on international deployment. The Musculoskeletal Screening Protocol (MSP) was used to collect prevalence data with respect to MSD, workload of previous work before preparation training and self-perceived health [12]. The questionnaire is self-administrated, and a physiotherapist was present during the survey to explain the procedure and to answer questions. This protocol was developed within the SwAF in the late 90s and has over the years been further adopted to suit different divisions of military personnel. The MSP was first published in 2008. The questions from this protocol that have been analyzed in the present study have not been changed from 2002 to 2012. The protocol includes questions regarding the one-year and point prevalence of musculoskeletal disorders (MSD), including pain, complaints and medically diagnosed injuries in ten anatomical parts (neck, upper back, low back, shoulders, elbow, hand, hip, knee, lower limb and foot), and if their MSD is experienced constantly or not with the options never, rarely or always. The protocol also contains a question about estimated workload in the respondent’s previous occupation, with the response options light/easy or heavy/hard. An additional five questions concerned perceived self-rated health, i.e. how the respondent experienced their physical body, mental health, social environment, physical environment and work ability. The response options were categorized as bad, good and excellent [5, 12, 15]. Both content and predictive validity of the protocol had been established [5, 12].

If soldiers report prevalence of MSD they were referred to the medical services for further examination and treatment.

Descriptive data, i.e. sex, age (presented in age groups 20–30, 31–40 and over 41 years) and body mass index (BMI) were also collected.

For statistical calculations, IBM SPSS Statistics, version 22, was used. Descriptive data are presented as number of (n), mean, standard deviation (SD), frequency (min-max), percentage (%) and 95% confidence interval (95% CI; based on the Wilson score interval) [16]. Data were checked for normal distribution before performing the statistical analysis.

Differences between cohorts were analyzed using the Chi-square test and Mann-Whitney’s *U* test statistics where the significant level was set at *p* < 0.05.

Variables with significant differences between groups were carried forward to logistic regression, with one-year prevalence of MSD in any body part as the outcome variable. Multiple logistic regression analysis was performed using a backward likelihood ratio model, where the final model only includes independently associated factors, with *p*-value set at < 0.05 for the outcome variable. The results are presented as odds ratios (OR) with 95% confidence interval (95% CI). Model fit was assured using the Hosmer-Lemeshow test, where a non-significant value (*p* > 0.05) indicates goodness-of-fit [17]. Collinearity between variables was investigated with the Spearman rank correlation coefficient.

## Results

### Participants

A total of 961 soldiers participated in the study, and out of these, 7% were women. The majority of the soldiers were between 20 and 30 years old. No clinically relevant differences were found between cohorts regarding height, weight and body mass index.

Significant differences between the two cohorts were found for stratified age groups, where 82% were between 20 and 30 years in 2002 and 67% in 2012 (*p* < 0.001). Significant differences existed between the cohorts in estimated workload in previous occupation (p < 0.001), i.e. before entering the preparation training for deployment, with soldiers in 2012 estimating a higher frequency of heavy/hard workload in their previous occupations. For more detailed information, see Table 1.

**Table 1:** Sex, age groups, estimated workload in previous occupation before entering preparation training presented as percentage (%) and body mass index (BMI, kg/m^2^) presented as mean, standard deviation (SD) for soldiers in Cohort 2002 and Cohort 2012 and significant differences between cohorts. Significant value *p* < 0.05

		Cohort		
		2002	2012	*p*-value
Sex				
Male	%	94	91	0.063
Female	%	6	9	
Age group (years)				
20–30	%	82	67	< 0.001
31–40	%	14	19	
41-	%	4	14	
BMI				
	Mean (SD)	24.6 (2.9)	25.0 (2.3)	0.013
Estimated workload pervious occupation				
Light/easy	%	73	46	< 0.001
Hard/heavy	%	27	54	

### Prevalence of MSD including pain and medically diagnosed injuries

In general, the prevalence of MSD was significantly higher among soldiers in the 2012 cohort compared to the 2002 cohort for both one-year prevalence and point prevalence for all anatomic locations. The knee was the most common anatomic location of MSD for both point and one-year prevalence in both cohorts; see Table 2.

**Table 2:** The one-year and point prevalence for musculoskeletal disorders (MSD) in ten body parts and for any body part in Cohort 2002 and Cohort 2012 presented in percentage (%) and 95% confidence interval (95% CI). *P*-value indicates the presence of any statistically significant difference between Cohort 2002 and Cohort 2012 regarding prevalence of MSD. Significant value *p* < 0.05

	Cohort 2002		Cohort 2012		
	n	Yes % (95% CI)	n	Yes % (95% CI)	*p*-value
One-year prevalence					
Neck	603	4.3 (2.9–6.3)	355	8.5 (6.0–11.8)	0.008
Upper back	603	3.8 (2.5–5.7)	355	10.4 (7.6–14.1)	< 0.001
Low back	603	6.0 (4.3–8.2)	355	18.9 (15.1–23.3)	< 0.001
Shoulders	603	3.6 (2.4–5.5)	354	11.0 (8.1–14.7)	< 0.001
Elbow	602	1.7 (0.9–3.1)	354	4.8 (3.0–7.6)	0.005
Hand	603	2.5 (1.5–4.1)	354	7.3 (5.0–10.6)	< 0.001
Hip	603	0.7 (0.2–1.8)	353	4.5 (2.8–7.3)	< 0.001
Knee	603	8.1 (6.2–10.6)	355	24.2 (20.0–29.0)	< 0.001
Lower leg	603	2.0 (1.1–3.5)	355	7.6 (5.3–10.9)	< 0.001
Foot	602	4.0 (2.7–5.9)	355	14.9 (11.6–19.0)	< 0.001
Any part	603	27.9 (24.4–31.6)	355	67.9 (62.8–72.6)	< 0.001
Point prevalence					
Neck	605	0.8 (0.3–2.0)	355	3.7 (2.1–6.2)	0.002
Upper back	604	0.2 (0.0–1.2)	355	5.6 (3.7–8.6)	< 0.001
Low back	605	0.8 (0.3–2.0)	354	5.9 (3.9–8.9)	< 0.001
Shoulders	604	0.3 (0.1–1.3)	354	6.2 (4.1–9.3)	< 0.001
Elbow	604	0.7 (0.2–1.8)	354	2.3 (1.1–4.5)	0.039
Hand	605	1.2 (0.6–2.4)	354	4.0 (2.4–6.6)	0.004
Hip	605	0.2 (0.0–1.2)	353	2.0 (0.9–4.1)	0.005
Knee	604	1.8 (1.0–3.3)	354	10.7 (7.9–14.4)	< 0.001
Lower leg	605	0.3 (0.1–1.3)	355	3.7 (2.1–6.2)	< 0.001
Foot	604	1.2 (0.6–2.4)	355	6.2 (4.1–9.2)	< 0.001
Any part	605	7.1 (5.3–9.5)	355	35.2 (30.4–40.3)	< 0.001

Further, a significant increase (*p* < 0.001) in how frequently the soldiers experienced their MSD was reported from 2002 to 2012, with 90.5% of soldiers in 2002 reporting that they rarely had MSD compared to 76.1% in 2012. In both cohorts, only 3% reported always experiencing the presence of MSD.

### Perceived health

**Fig. 1:**
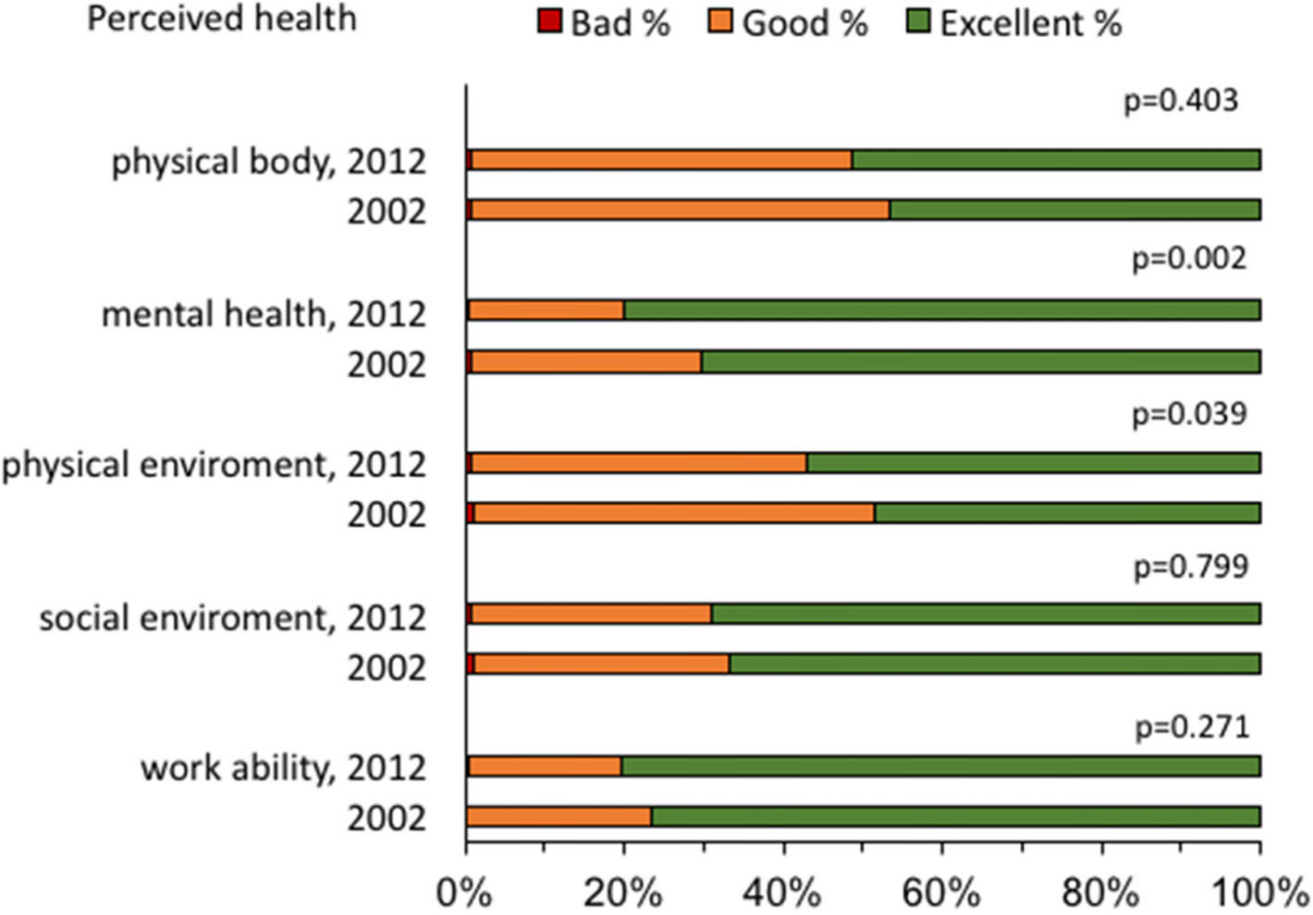
Perceived health in Cohort 2012 and Cohort 2002 categorized as bad, good and excellent. *P*-value between cohorts regarding perception of physical body, mental health, physical environment, social environment and work ability. Significant value *p* < 0.005

Most soldiers reported good to excellent perceived health, i.e. the perception of the physical body, mental health, physical and social environment, and work ability. Although in general, soldiers in 2012 reported higher scores than those in 2002, they did not reach significance except for two of the five areas, see Fig. 1. That is, significant differences between cohorts were found with respect to how they perceived their physical environment (*p* = 0.039) and mental health (*p* = 0.002), with more favorable responses for both found in 2012.

### Factors associated with prevalence of MSD

In the final logistic regression model, the odds of reporting one-year prevalence of MSD in any body part were 5.28 times higher for soldiers in 2012, 1.91 times higher in age group 31–40 and 2.84 times higher in age group 41 and above. A decrease in the odds was seen among soldiers who rated their physical body as excellent (OR 0.64). See Table 3.

**Table 3:** Multiple logistic regression model; initial and final model for factors associated with one-year prevalence of musculoskeletal disorders (MSD). Odds ratio (OR), 95% confidence interval (CI) of odds ratio and *p*-value. Significant level *p* < 0.05. Goodness-of-fit of the model according to Hosmer-Lemenshow test [17]

		Initial model				Final model			
	n	OR	95% CI		*p*-value	OR	95% CI		*p*-value
	766		Lower	Upper			Lower	Upper	
Mission									
2002	520	1.00							
2012	246	4.91	3.46	6.98	< 0.001	5.28	3.77	7.40	< 0.001
Gender									
men	709	1.00							
women	57	1.75	0.93	3.29	0.083				
Age group (years)									
20–30	600	1.00							
31–40	113	1.88	1.21	2.94	0.005	1.91	1.23	2.96	0.004
41-	53	2.48	1.28	4.82	0.007	2.84	1.48	5.45	0.002
BMI	766	1.02	0.96	1.09	0.456				
Workload									
light/easy	494	1.00							
hard/heavy	272	1.22	0.86	1.72	0.265				
Perceived health									
Physical body									
bad	5	1.67	0.22	12.44	0.619				
good	401	1.00							
excellent	360	0.65	0.47	0.89	0.008	0.64	0.46	0.88	0.006

Hosmer-Lemeshow test *p* = 0.95

## Discussion

The present study found that the prevalence of musculoskeletal disorders (MSD) has significantly increased from 2002 to 2012 among Swedish soldiers preparing for international missions. MSD were most common in the knee, low back and foot. Moreover, MSD in the present study were associated with deployment in Cohort 2012 and with older age.

Larsson et al. [5] examined 469 male soldiers during their basic military training and found consistent findings with respect to the highest prevalence of MSD in the low back, knee and foot, as reproduced in the present study. The low back is an area with high prevalence of MSD among soldiers, and could be explained by heavy physical exposure when carrying heavy packing/equipment, long marches and long periods of sitting with combat gear in a non-ergonomic position, for example in combat vehicles. This is in accordance with the findings of Nissen et al. [18], who found that old age, lack of support from leaders, psychological stress, awkward working positions and working in storehouses or depots were significantly associated with low back pain. The high prevalence of MSD in the low back has earlier been confirmed in a study of US soldiers serving in Afghanistan [7]. Both the present and previous studies have also found high prevalence of MSD in the lower extremities, and possible explanations for this may be that soldiers are exposed to tasks involving heavy physical loads and carrying heavy equipment during long marches.

In the present study, soldiers in Cohort 2002 had an MSD point prevalence of 7.1% compared to 35.2% in 2012, highlighting the immense increase in MSD prevalence over 10 years. Furthermore, the one-year prevalence of MSD showed a similar increase, with a proportion of 27.9% in 2002 compared to 67.9% in 2012. These findings demonstrating the increased prevalence of MSD are corroborated by almost an entire body of literature within military populations [8, 13].

Roy et al. argue that the main cause behind the increased prevalence of MSD is heavier equipment and previous complaints or injuries among many soldiers, while the heavy load can cause new associated problems [7]. In the present study, no measures of carried equipment were conducted, which should be taken into consideration when planning and performing future studies. Previous studies have found a correlation between the load of carried equipment and prevalence of MSD [8, 14]. Further, another explanation for the increased prevalence of MSD within the SwAF could be the increasing age of the soldiers. It is known that the incidence of MSD increases with age [11], which is supported by results from the present study with a higher odds ratio found for soldiers > 30 years of age. Further explanations could be that an older population of soldiers may have been exposed to heavy physical loads over a longer time period as well as having prior experience of MSD. Both of these aspects may increase the risk of developing new MSD [19]. In 2010, Sweden’s policy changed from compulsory national service to a regular army. This development has led to an increase in the average age of soldiers. During the compulsory national service, many persons ended their time within the SwAF after conscription. Today, however, soldiers more commonly serve for a longer period, up to approximately 8 years, often including deployment. Taken together, the increased average age of soldiers along with serving a longer time may partly explain the increased prevalence of MSD.

Screening of soldiers’ perceived health can provide indications of how individuals will cope with various stress factors in their work. Frequent stress lasting over a longer period, along with the lack of time for recovery, has been found to increase the risk of injury. In the present study, soldiers in 2012 reported better self-perceived health than soldiers in 2002. Other studies within the armed forces found similar results regarding self-perceived health [6]. Although the 2012 cohort reported a higher prevalence of MSD, they had better self-perceived health in comparison with the 2002 cohort. Campbell et al. [20] suggest that there are several components that contribute to self-perceived health, such as personality, experience and previous mental health.

Another reason for the increased prevalence of MSD could relate to the more frequent international deployment over the last 10 years, which is in line with several studies reporting the increasing physical load during international deployments [7, 13].

The present study did not find significant differences between MSD and the experience of their own working ability. Similar findings were reported by Glad et al. [6], who found that despite experiencing complaints, none of the soldiers perceived reduced working capacity/ability. On the contrary, Roy et al. [7] found a correlation between those with complaints and perceived reductions in working ability. As a part of the process of improving working conditions and environment as well as decreasing the prevalence of MSD, it is imperative to identify the soldier’s individual capacity and ability to adapt to the working demands. This stresses the importance of defining work requirements for various roles within the SwAF and work capacity among military personnel. One suggestion, perhaps, could be to adapt and increase physical load gradually while ensuring individualized progression from the beginning of training [12, 21]. This suggestion not only has the potential to improve the soldiers’ health by reducing personal suffering, but also addresses economic losses to the individual, organization and society in the form of sick leave and disability pension. It is therefore of importance to monitor the prevalence of MSD in a long-term perspective. It is, however, possible to vary the load and the demands of physical capacity at a later stage when role-specific training and further services commence. In response to this, the SwAF currently implement a preventive interventions program with the aim of optimizing the individual’s physical and mental health. The program also aims to optimize performance before, during and after deployment [12].

One limitation in the present study relates to the soldiers reporting previous experiences of MSD when attempting to establish one-year prevalence. Therefore, recall bias with respect to remembering complaints and injuries, as well as the extent and duration of MSD, could not be eliminated, as previously discussed by Carragee et al. [22]. The sample size of the present study was rather large, which could have led to statistically significant differences of a small variation in pertinent outcomes, changes/variation otherwise explained by the natural variance of the measure (method errors). To reinforce statistically significant differences, the 95% confidence interval was used to evaluate the magnitude of change [23]. The results from the present study can be generalized to soldiers with similar positions in Sweden, but could also be extended to other countries with a similar military system and working requirements, such as other Scandinavian countries In addition, other agencies with similar physical workloads and work requirements, such as police and firefighters, could benefit from the knowledge generated in this study.

## Conclusion

The prevalence of MSD increased remarkably over a ten-year period among Swedish soldiers preparing for international missions. With increasing age as one risk factor, systematic monitoring of MSD throughout the soldiers’ careers and implementation of targeted primary-to-tertiary preventive programs are thus important.

## Data Availability

There are ethical restrictions regarding data availability for public release in this study since identification of participants from the data cannot be ruled out. Data contained in this paper are considered as sensitive. According to the Ethical committee in Sweden, and within the Swedish Armed Forces, we are not allowed to have data available for public release due to ethical restrictions. We can only make the data available upon reasonable request, which will also involve discussions with the Swedish Armed Forces. Contact information: Swedish Armed Forces Research coordinator Anders Claréus 107 85 Stockholm/Sweden Phone: + 46 8 788 85 26 E-mail: anders.clareus@mil.se

## Abbreviations

BMI: Body mass index
CI: Confidence interval
MSD: Musculoskeletal disorders
MSP: Musculoskeletal Screening Protocol
OR: Odds ratio
SD: Standard deviation
SwAF: Swedish Armed Forces

## References

[1] Taanila H, Suni JH, Kannus P, Pihlajamaki H, Ruohola JP, Viskari J, Parkkari J (2015). Risk factors of acute and overuse musculoskeletal injuries among young conscripts: a population-based cohort study. BMC Musculoskelet Disord.

[2] Nissen LR, Guldager B, Gyntelberg F (2009). Musculoskeletal disorders in main battle tank personnel. Mil Med.

[3] Zambraski EJ, Yancosek KE (2012). Prevention and rehabilitation of musculoskeletal injuries during military operations and training. J Strength Cond Res.

[4] Monnier A, Djupsjobacka M, Larsson H, Norman K, Ang BO (2016). Risk factors for back pain in marines; a prospective cohort study. BMC Musculoskelet Disord.

[5] Larsson H, Broman L, Harms-Ringdahl K (2009). Individual risk factors associated with premature discharge from military service. Mil Med.

[6] Glad D, Skillgate E, Holm LW (2012). The occurrence and severity of musculoskeletal disorders in Swedish military personnel during peacekeeping operations in Afghanistan. Eur Spine J.

[7] Roy TC, Knapik JJ, Ritland BM, Murphy N, Sharp MA (2012). Risk factors for musculoskeletal injuries for soldiers deployed to Afghanistan. Aviat Space Environ Med.

[8] Kaufman KR, Brodine S, Shaffer R (2000). Military training-related injuries: surveillance, research, and prevention. Am J Prev Med.

[9] Monnier A, Larsson H, Djupsjobacka M, Brodin LA, Ang BO (2015). Musculoskeletal pain and limitations in work ability in Swedish marines: a cross-sectional survey of prevalence and associated factors. BMJ Open.

[10] Punnett L, Wegman DH (2004). Work-related musculoskeletal disorders: the epidemiologic evidence and the debate. J Electromyogr Kinesiol.

[11] da Costa BR, Vieira ER (2010). Risk factors for work-related musculoskeletal disorders: a systematic review of recent longitudinal studies. Am J Ind Med.

[12] Larsson H (2009). Premature discharge from military service: risk factors and preventive interventions: Diss.

[13] Orr RM, Coyle J, Johnston V, Pope R (2017). Self-reported load carriage injuries of military soldiers. Int J Inj Control Saf Promot.

[14] Knapik JJ, Reynolds KL, Harman E (2004). Soldier load carriage: historical, physiological, biomechanical, and medical aspects. Mil Med.

[15] Larsson H, Tegern M, Harms-Ringdahl K (2012). Influence of the implementation of a comprehensive intervention programme on premature discharge outcomes from military training. Work.

[16] Brown L, Cai TT, DasGupta A. Interval estimation for a binomial proportion Statist Sci 2001;16:101–117.

[17] Hosmer DJ, Lemeshow S, Sturdivant RX. Applied logistic regression. 3rd ed. Hoboken: Wiley; 2013.

[18] Nissen LR, Marott JL, Gyntelberg F, Guldager B (2014). Deployment-related risk factors of low back pain: a study among Danish soldiers deployed to Iraq. Mil Med.

[19] Knapik JJ, Jones SB, Darakjy S, Hauret KG, Nevin R, Grier T, Jones BH (2007). Injuries and injury risk factors among members of the United States Army band. Am J Ind Med.

[20] Campbell D, Nobel OB-Y (2009). Occupational stressors in military service: a review and framework. Mil Psychol.

[21] Larsson H, Tegern M, Monnier A, Skoglund J, Helander C, Persson E, Malm C, Broman L, Aasa U (2015). Content validity index and intra- and inter-rater reliability of a new muscle strength/endurance test battery for Swedish soldiers. PLoS One.

[22] Carragee EJ, Cohen SP (2009). Lifetime asymptomatic for back pain: the validity of self-report measures in soldiers. Spine (Phila Pa 1976).

[23] Chan YH (2003). Biostatistics 101: data presentation. Singap Med J.

